# Percolation Theory Reveals Biophysical Properties of Virus-like Particles

**DOI:** 10.1021/acsnano.1c01882

**Published:** 2021-07-23

**Authors:** Nicholas E. Brunk, Reidun Twarock

**Affiliations:** † Wolfram Research, Champaign, Illinois 61820, United States; ‡ VeriSIM Life, San Francisco, California 94104, United States; ¶ Intelligent Systems Engineering, Indiana University, Bloomington, Indiana 47408, United States; § Departments of Mathematics and Biology, York Cross-disciplinary Centre for Systems Analysis, University of York, York YO10 5GE, U.K.

**Keywords:** virus-like particle, virus disassembly, percolation theory, virus nanotechnology, generalized quasi-equivalence principle

## Abstract

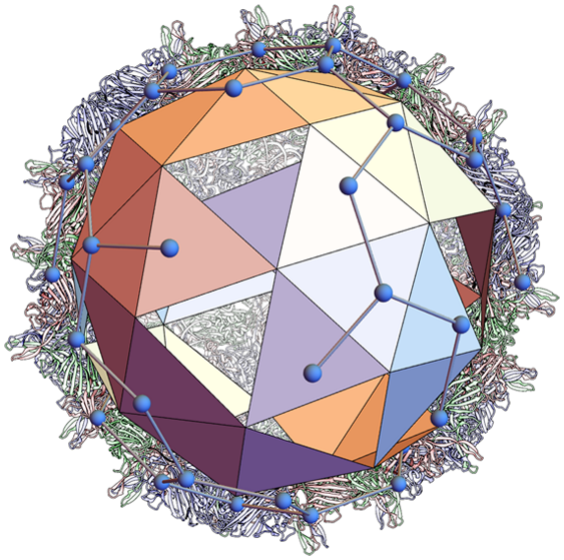

The viral protein containers that encapsulate a virus’ genetic material are repurposed as virus-like particles in a host of nanotechnology applications, including cargo delivery, storage, catalysis, and vaccination. These viral architectures have evolved to sit on the knife’s edge between stability, to provide adequate protection for their genetic cargoes, and instability, to enable their efficient and timely release in the host cell environment upon environmental cues. By introducing a percolation theory for viral capsids, we demonstrate that the geometric characteristics of a viral capsid in terms of its subunit layout and intersubunit interaction network are key for its disassembly behavior. A comparative analysis of all alternative homogeneously tiled capsid structures of the same stoichiometry identifies evolutionary drivers favoring specific viral geometries in nature and offers a guide for virus-like particle design in nanotechnology.

## Introduction

Mining design features from nature is a cornerstone of virus nanotechnology. The molecular designs of the viral protein shells, called viral capsids, which surround and thus protect the viral genomes, are exploited as virus-like particles (VLPs) for cargo delivery and storage.^1,2^ Their physical properties can be tuned to support the desired purposes,^3−6^ for example, by modulating the charge of particles *via* peptide binding or through direct removal and replacement of protein subunits as a means of functionally decorating the capsid surface.^7,8^ The net charge is also important for a variety of viral mechanisms, including assembly and the encapsidation of charged polymers.^9−12^ Perforated capsids^7,13^ are reminiscent of a breadboard with modular components. They offer the opportunity to fractionally refill the VLP surface in order to modulate its steric, elastic, and electrostatic properties.^2,8^ Modular VLP technologies depend crucially on a VLP’s resistance to fragmentation upon subunit removal prior to their replacement. Much like the shape of building blocks would affect the disassembly of a tower, as in the board game Jenga,^14^ the shape and valency of the capsid building blocks (capsomers) affect the assembly and disassembly behavior of the virus. A capsid’s geometric design and its implied subunit-bond network are therefore essential for its role in cargo encapsidation and release.

The number of distinct viral capsid designs in nature is limited. This is a consequence of the principle of genetic economy,^15^ which stipulates that viral capsids are formed from multiple copies of identical protein subunits synthesized from the same genomic fragment, thus reducing the overall length of the genomic sequence required to code for the capsid. As identical protein subunits form the same types of local interactions across the capsid surface, they self-assemble into capsids with a high degree of symmetry, and icosahedral viruses make up the vast majority of particle architectures in the virosphere. Caspar and Klug’s quasi-equivalence theory models icosahedral viral capsid architecture *via* polyhedral blueprints, which are parametrized in terms of the triangulation number *T*,^16^ implying that capsids must be formed from precisely 60*T* protein subunits. The cancer-causing papillomaviruses, however, form a notable exception, and prompted the generalization of their theory in Viral Tiling theory.^17^ Recently, a comprehensive theory of viral capsid architecture has been introduced based on a generalized quasi-equivalence principle^18^ that includes both Caspar–Klug theory and Viral Tiling theory as special cases.

We analyze here the impact of distinct capsid designs in this classification on capsid disassembly. The importance of the subunit-bond network topology for the resistance of the capsid to fragmentation and disassembly has been demonstrated previously for Hepatitis B virus (HBV) *in vitro*. In these experiments, subunits were removed from intact capsids, or from capsids in which chemical cross-linking prevented the removal of passivated subunits, using denaturant. Single particle mass spectrometry was used to interrogate the resulting particles, revealing a marked absence of any particles fewer than 90 protein dimers, in excellent agreement with the theoretically predicted fragmentation threshold.^7,19^ However, the dependence of capsid disassembly on capsid geometry and topology has not been investigated before. We are closing this gap here by developing a generalized percolation theory for virus capsid disassembly. These fragmentation thresholds characterize the onset of dissociation of the capsid in terms of the numbers of subunits removed or of the intersubunit contacts broken. They are stricter versions of the traditional percolation thresholds, which characterize the subsequent, abrupt disappearance of all clusters of the order of system size. We investigate how the topologies of alternative capsid geometries with identical protein stoichiometries and their associated subunit-bond networks impact the VLP fragmentation as subunits are randomly removed or when bonds are randomly broken. In particular, the maximal number of subunits (or, respectively, bonds) that may be removed without, on average, inducing fragmentation of the remaining capsid shell (*i.e.*, the subunit and bond fragmentation thresholds) are computed, enabling a comparative analysis of the resilience to fragmentation of distinct capsid designs. Our results reveal the mechanistic pressures on viral evolution and provide a possible explanation for the abundance of specific viral capsid designs in nature. They also inform VLP design in bionanotechnology applications.

## Results and Discussion

### Graphs as Topological Descriptors of Viral Capsids

**Figure 1:**
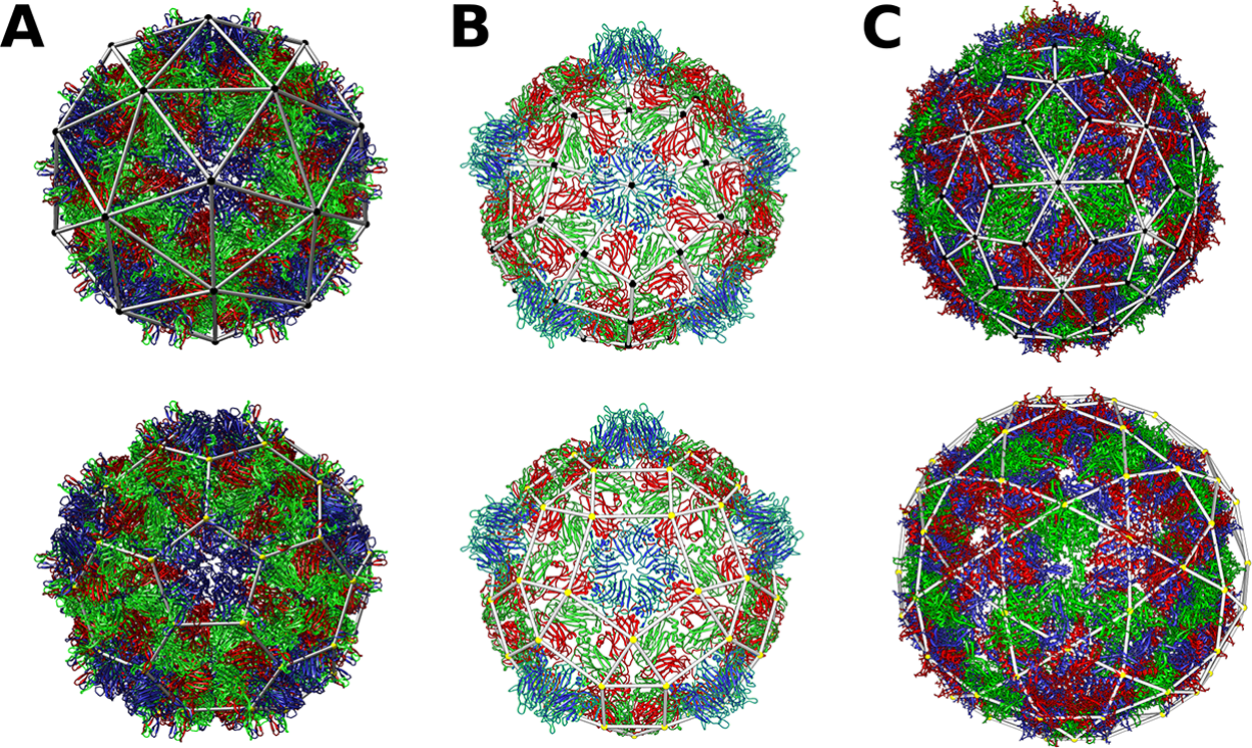
Protein layouts and interaction networks for different capsid geometries. There are three distinct types of structural organization in the viral capsids that are classed as *T* = 3 architectures in Caspar Klug theory: (A) the triangular tiling in which each subunit is trivalent, as is the case for Pariacoto virus (1f8v); (B) the kite-shaped tiling in which each subunit is tetravalent, as is the case for Tobacco Ring virus (1a6c); (C) the rhombic tiling in which each subunit is tetravalent, as is the case for bacteriophage MS2 (2ms2). Underneath are shown the dual tilings superimposed on the capsids, which encode the topology of the interaction network.

Viral capsid architectures are modeled in terms of surface tessellations termed tilings, in which tiles indicate capsid building blocks (capsomers). For the smallest icosahedral capsids formed from 60 copies of a single capsid building block, there is only one possible triangular layout that is fully determined by icosahedral symmetry. The next larger capsids according to Caspar and Klug theory have triangulation number *T* = 3 and are formed from 60*T* = 180 proteins. These include the architectures of plant viruses, such as Cowpea Chlorotic Mottle virus (CCMV), that are used in virus nanotechnology.^20^ According to the classification of virus architecture based on the generalized quasi-equivalence principle,^18^ there are three possible, topologically distinct layouts for capsids with this stoichiometry. Two of them correspond to tilings formed from 60 tiles, each representing three protein subunits: a triangular tile as seen in Pariacoto virus (1f8v, Figure 1A top), and a kite-shaped tile as in Tobacco Ringspot virus (1a6c, Figure 1B top). In addition, there is a capsid design formed from 90 rhombic tiles, each representing two protein subunits, as seen in bacteriophage MS2 (2ms2, Figure 1C top). These capsids are formed from one type of protein subunit, but these subunits can be in distinct conformations. Icosahedral symmetry constrains their positions in the capsid but allows for up to three distict conformers in a *T* = 3 architecture. As a consequence, the interfaces between tiles are not identical. Each triangular and kite tile accommodates proteins in three distinct conformations, and rhomb tilings are formed from two distinct types of protein dimer, a symmetric, and an asymmetric one, which are composed of protein subunits in different conformations.

The intersubunit bond network, *i.e.*, the topology of the network describing the contacts between adjacent subunits, corresponds to the dual tiling (Figure 1, bottom row), with vertices *V*_0_ (yellow) representing individual tiles (capsomers), and edges *E*_0_ interfaces between adjacent tiles. We use the graph  corresponding to the dual tiling as a topological descriptor of the capsid. The vertices and edges form sets from which *i* elements may be randomly removed in order to probe the capsid’s resilience to fragmentation using percolation theory. As in previous work, we refer to a complete capsid as  and any partially disassembled capsid randomly missing *i* tiles (vertices *v*) as .^19^ The fraction of deleted vertices is thus *f*_*v*_^*d*^ = *i*/*V*_0_ and the fraction remaining is *p*_*i*_^*v*^ = 1 – *f*_*v*_^*d*^, both of which are symmetric common variables in percolation theory. We use here the fraction deleted, *f*_*v*_^*d*^. Similarly, we refer to a capsid with *j* randomly broken bonds as *G*_*j*_^*e*^ and denote the fraction of broken bonds (edges *e*) as *f*_*e*_^*d*^ = *j*/*E*_0_. Note that the perforated capsid *G*_*i*_^*v*^ (*G*_*j*_^*e*^) can fragment into two or more separated clusters. These are then referred to as distinct “connected components” following terminology in graph theory.

### Percolation Theory for Viral Capsids

Capsid fragmentation occurs *via* any dissociation of single units (tiles) or smaller clusters from the bulk of the capsid and is followed quickly by complete dissociation and breakdown of the remaining long-range connectivity. In order to profile this behavior, we have numerically determined the subunit and bond inverse fragmentation thresholds (*f*_*T*_) as an ensemble average over different stochastic simulations for each of the three geometrically and topologically distinct *T* = 3 capsid architectures shown in Figure 1.

**Figure 2:**
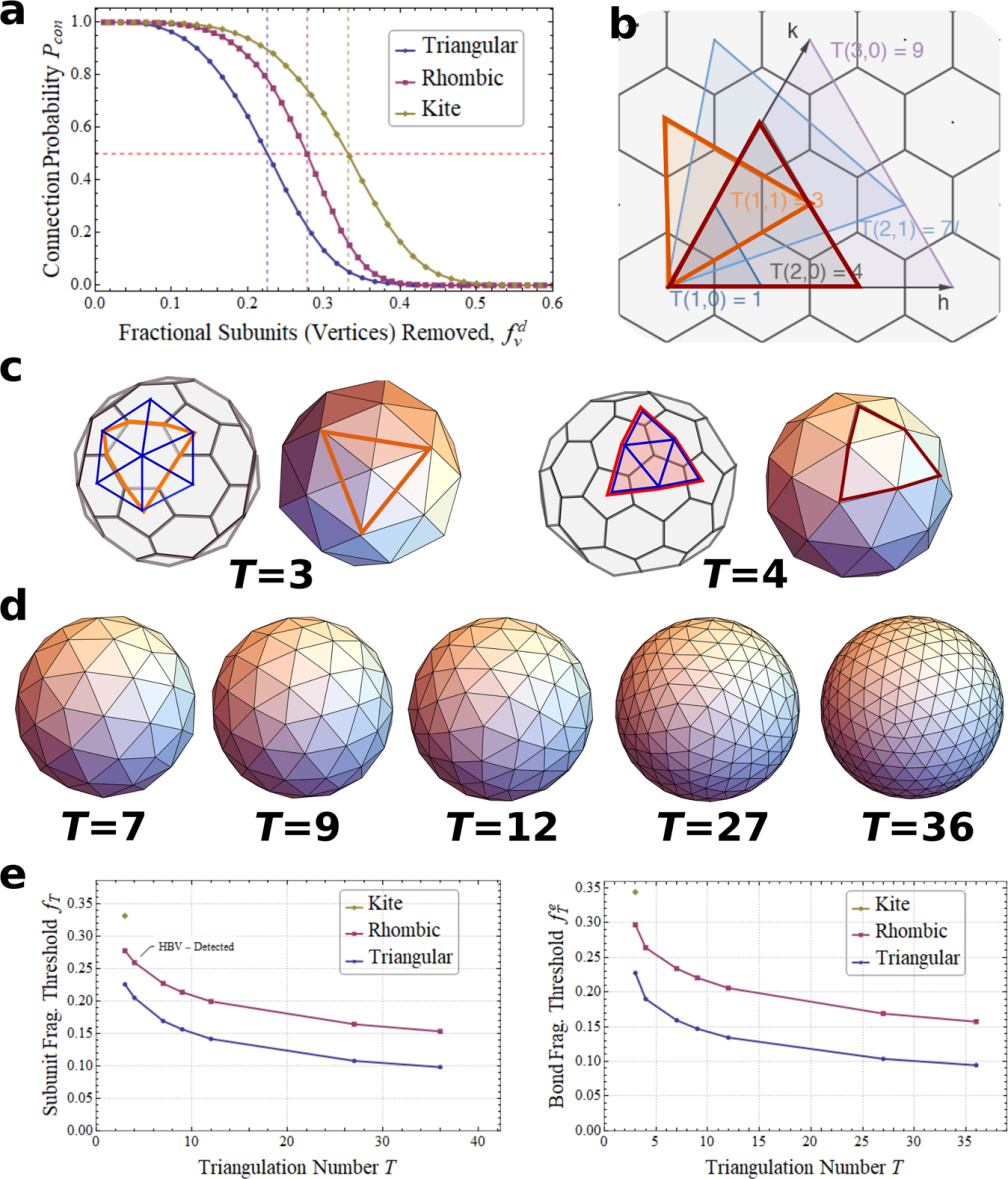
Some viral architectures are less stable and more prone to fragmentation. (a) The rapidly decaying, sigmoidal probability, *P*_*con*_, that a given *T* = 3 architecture is still connected, as a function of the fraction *f*_*v*_^*d*^ of subunits removed, computed by averaging 100,000 Monte Carlo replicates per data point. The fragmentation threshold of each viral blueprint is indicated by gridlines. (b) The *T*-number construction for virus tilings according to Caspar and Klug.^16^ An equilateral triangle, representing one of the 20 triangular faces of an icosahedron, is embedded into a hexagonal lattice such that the corners of the triangle are located in the centers of hexagons. The triangles corresponding to a *T* = 3 and *T* = 4 embedding are shown in orange and red, respectively. (c) The spherical architecture corresponding to the *T* = 3 and *T* = 4 embeddings are shown together with their corresponding dual triangulations to their right; the orange, and respectively red, triangles from (b) are shown superimposed. (d) Structures of the homogeneously tiled virus architectures, for which subunit and bond fragmentation thresholds (*f*_*T*_ and *f*_*T*_^*e*^) have been computed (Table 1), demonstrating their dependence on capsid size. (e) The subunit (left) and bond (right) fragmentation thresholds for selected kite, rhombic, and triangular tilings of sizes up to *T* = 36. The subunit fragmentation threshold for rhombic *T* = 4 tilings has already been experimentally confirmed for HBV;^7,19^ others remain to be observed. The kite tiling is the most stable, followed by rhombic tilings, while triangular tilings are the most prone to fragmentation.

The predicted probability *P*_*con*_(*f*_*v*_^*d*^) that a capsid missing a given fraction of subunits will remain connected shows sigmoidal decay (Figure 2a), revealing a fairly abrupt transition from an intact, perforated capsid to dissociated fragments. The point at which the capsid is, on average, fragmented corresponds to the fragmentation threshold *f*_*T*_, defined by *P*_*con*_(*f*_*v*_^*d*^) = 0.5. Notably, the *f*_*T*_ of the triangular, kite, and rhombic *T* = 3 architectures are distinct, with values of *f*_*T*_ = (0.226, 0.331, 0.278), respectively, suggesting that the corresponding capsids exhibit different propensities for fragmentation. We converted the traditional percolation thresholds for the infinite planar lattices, from which our spherical lattices have been derived, into deletion fractions, *p*_*c*_ ≈ (0.303, 0.378, 0.347),^21−24^ to make them directly comparable to the fragmentation thresholds computed here. We note that the relative values are similar for the different lattice types, with the triangulation scoring lowest, and the kite tiling highest. Differences in absolute values are likely due to the fact that we are penalizing against the exclusion of singlets. Our results are intuitive: the fragmentation threshold *f*_*T*_ (and percolation threshold) of a given lattice scales with the subunit valency or number of bonds per subunit, *i.e.*, *f*_*T*_ ∝ *k*_0_, where . Indeed, we observe successively higher thresholds in the tetravalent (4-connected) graphs associated with the rhombic and kite tilings, than in the graph of the trivalent (3-connected) triangular tiling.

**Table 1:** Fragmentation Thresholds *f*_*T*_ and *f*_*T*_^*e*^ for Different Tiling Types and Sizes

tiling type	*T*-number	*f*_*T*_	*f*_*T*_^*e*^
	3	0.226	0.228
	4	0.205	0.19
	7	0.169	0.159
triangular	9	0.156	0.147
	12	0.142	0.134
	27	0.108	0.104
	36	0.098	0.094
	3	0.278	0.297
	4	0.26	0.264
	7	0.227	0.234
rhombic	9	0.214	0.221
	12	0.199	0.206
	27	0.164	0.169
	36	0.153	0.157
kite	3	0.331	0.344

The kite tiling is the most stable of the three homogeneously tiled capsid types with respect to random subunit removal and is the only one that is resistant to fragmentation beyond 40% subunit removal (*f*_*v*_^*d*^ = 0.40). This is mirrored by the bond fragmentation threshold based on random breakage of bonds rather than removal of tiles, which is *f*_*T*_^*e*^ = (0.208, 0.318, 0.288) for the triangular, kite, and rhombic tiling, respectively. We again compare with the standard percolation thresholds, *p*_*c*_^*e*^ = (0.347, 0.475, 0.476).^21−24^ We observe that in both cases the triangulation has the lowest value, whereas the two four-coordinated tilings, the kite and rhombic tiling, both have higher ones. We note that in the spherical tilings, there is a distinction between the fragmentation thresholds of these two four-coordinated tilings that is not visible in the percolation thresholds of the corresponding planar lattices. Interestingly, regarding initial fragmentation, the kite tiling remains the most stable tiling also with respect to random bond breakage.

We repeated the same construction for larger polyhedra that can be derived from the same planar lattices following the procedure introduced by Twarock and Luque.^18^ For the hexagonal lattice, the corresponding polyhedra are known as Goldberg polyhedra or geodesic icosahedra. They are classified in terms of the triangulation number^16^ (T-number) *T*, which specifies the position of one triangular face of an icosahedron in a hexagonal lattice embedding (Figure 2b). Each triangle is fully determined by *T* = *h*^2^ + *hk* + *k*^2^, with two integer numbers *h* and *k*. The latter represent steps between midpoints of adjacent hexagons along two vectors *h⃗* and *k⃗*, respectively, that intersect at the midpoint of a hexagon at an angle of π/3, cutting through two adjacent edges. The examples of *T* = 3 and *T* = 4 architectures are shown in Figure 2c. The Goldberg polyhedra have 12 pentagonal and 10(*T* – 1) hexagonal faces.^16^ Their duals, triangulations called deltahedra, are classified in terms of *T*, which indicates how many facets tile, by area, one of the 20 triangular faces of the icosahedral reference frame. These polyhedra have 20*T* triangular facets and 30*T* edges (bonds). A similar approach based on the trihexagonal lattice results in rhombic tilings formed from 30*T* rhombic faces and 60*T* bonds.^18^ The subunit and bond *f*_*T*_s for homogeneously tiled capsids up to *T* = 36 are provided in Table 2, demonstrating that this trend also persists for larger capsids. The tiling type, or capsomer geometry, is thus a determinant of robustness against disassembly.

**Table 2:** Parameters Directly Relevant to Our Percolation Theory Model

parameter	definition
*k*_0_	initial capsid tiling’s subunit valency (edges per vertex)
*f*_*v*_^*d*^	fraction of vertices/subunits deleted (independent variable)
*f*_*e*_^*d*^	fraction of edges/bonds deleted (independent variable)
*p*_*V*_	fraction of vertices/tiles remaining
*p*_*E*_	fraction of edges/bonds remaining
*P*_*con*_	probability remaining subunits are connected (dependent variable)
*f*_*T*_	vertex fragmentation threshold (where *P*_*con*_(*f*_*v*_^*d*^) = 0.5)
*f*_*T*_^*e*^	bond fragmentation threshold (where *P*_*con*_(*f*_*e*_^*d*^) = 0.5)
*p*_*c*_	vertex percolation threshold
*p*_*c*_^*e*^	bond percolation threshold

### Implications for Capsid Stability

In previous work, we introduced a closed-form empirical equation for general graphs, quantifying capsid stability in terms of the fraction *f*_*v*_^*d*^ of randomly removed vertices. It characterizes the fraction of remaining bonds (edges) *p*_*E*_ as a function of the remaining fraction of subunits (or vertices) *p*_*V*_ = 1 – *f*_*v*_^*d*^:
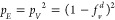
1Note that subunit valency is not explicitly contained in this equation. Thus, differing fragmentation thresholds between tilings correspond to different fractions of preserved bonds at each threshold, implying that the impact of capsid geometry on stability extends beyond bond valency differences in the corresponding intersubunit bond networks. Indeed, while higher subunit valency enhances capsid stability and increases the fragmentation threshold, fragmentation appears to also be size-dependent. This can be seen from the following argument. The *T* = 3 kite tiling has 60 tetravalent subunits and according to our analysis is more stable than the tetravalent 90-subunit rhombic tiling. However, for any given *T*-number, there are consistently 50% more subunits in the rhombic tilings, so that the ratio of building blocks alone cannot explain the shift in the fragmentation threshold seen in Figure 2e, where the values of the subunit (and bond) fragmentation thresholds are plotted against capsid size (in terms of the *T*-number). This implies that the stability difference between the rhombic and kite tilings is due to both the differing topology and the dependence of the subunit (and bond) fragmentation thresholds on capsid size.

The observed size dependence is, in part, due to an increase in the potential for exclusion of small clusters and single subunits from the bulk of the capsid: as the number of subunits increases, so does the probability of excluding small clusters. However, this size dependence appears to plateau at a value determined by the topology of the lattice. The triangular tiling, having lower subunit valency, is less resilient to fragmentation, particularly at higher *T*-numbers. By contrast, fragmentation of a rhombic tiling at *T* = 36 requires removal of a ≈50% larger fraction of its subunits than for a triangular tiling, while it requires only a ≈20% larger fraction to disassemble a *T* = 3 rhombic tiling over a triangular one. Given the need for viral capsids to provide adequate protection for the genome between rounds of infection, this may explain the increasing relative abundance of rhombic tilings in nature as the size of the virus (in terms of its *T*-number) increases. While there are examples of triangular and rhombic tilings at *T* = 4, rhombic tilings dominate from *T* = 7.

### Fragment Size Distributions

**Figure 3:**
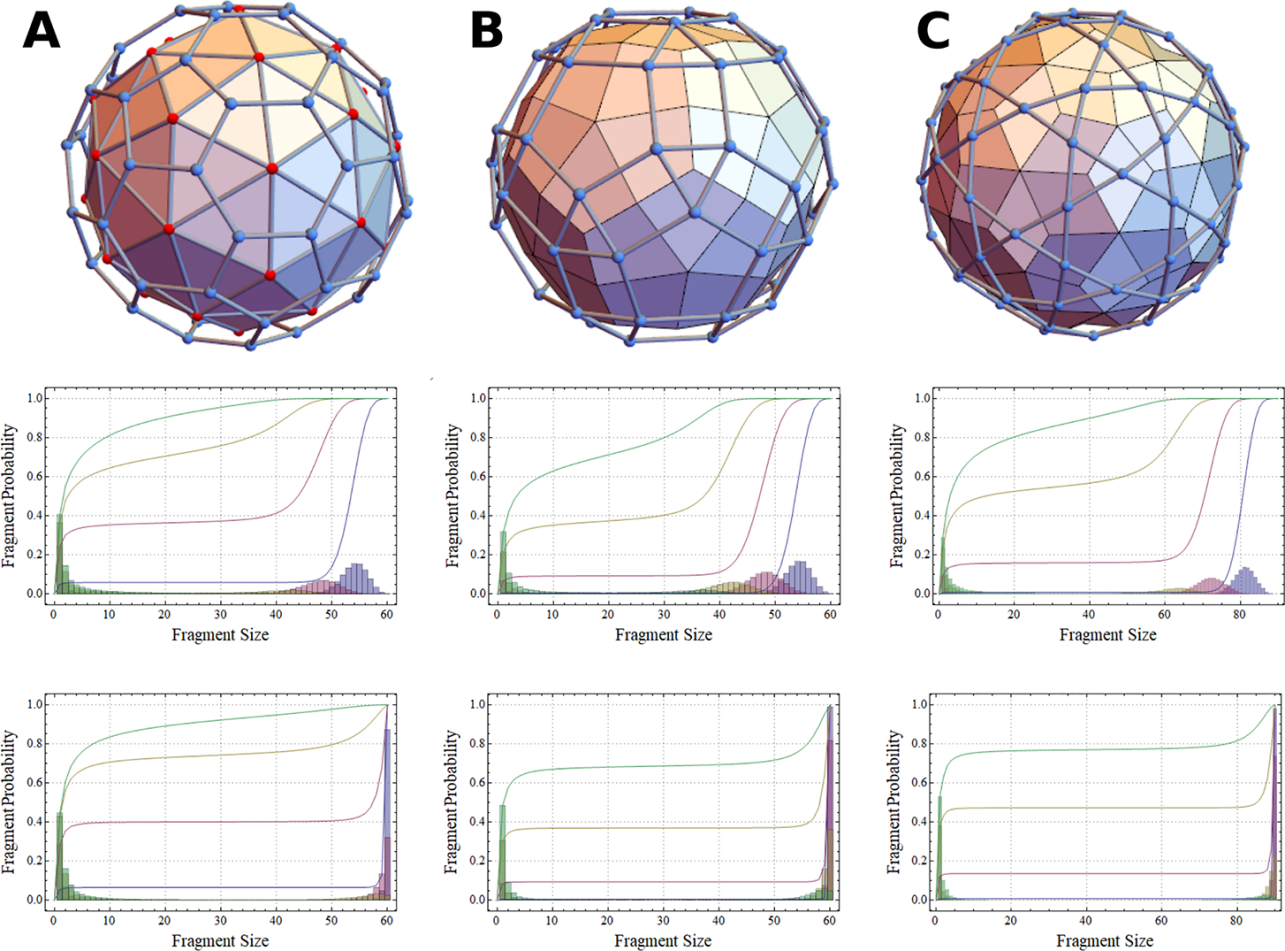
Fragment size distributions for different capsid types: (a) triangular, (b) kite, and (c) rhombic tiling design. Middle and bottom row: The histogram probability density functions (PDFs) of fragments of various sizes with normally distributed *f*_*v*_^*d*^ corresponding to 10% (blue), 20% (purple), 30% (gold), and 40% (green) subunit removal from the respective capsids/tilings (middle row) and edge removal from the corresponding interaction networks (bottom). These curves exclude the explicitly removed subunits and are overlaid with the cumulative distribution functions (CDFs), the slope of which indicates the fraction of species of that size. Interestingly, random bond breakage does not exhibit the same gradual decrease in fragment size until complete dissociation, as is observed experimentally for the dissociation of passivated subunits. Our theory predicts abrupt fragmentation beyond the fragmentation threshold and identifies expected concentrations that are experimentally measurable.

A capsid disassembly experiment, such as that implemented with HBV, is expected to result in a distribution of perforated viruses missing a mean number *i* (or fraction *f*_*v*_^*d*^) of their subunits.^7^ The precise numbers of distinct products are determined by experimental conditions such as denaturant concentration or the relative ratio of regular versus passivated subunits.^7,19^ In regimes sufficiently below the fragmentation threshold *f*_*T*_, this results in a binomial distribution of clusters centered around the mean number of removed subunits *i*. Before taking into account thermodynamic and kinetic effects that may skew this distribution, a greater topologically induced shift will occur due to significant fragmentation at, and just beyond, *f*_*T*_. Upon fragmentation, the binomial cluster size distribution will break down rapidly as singlets and small clusters dissociate from the larger whole, followed by complete disassembly. This can be seen in Figure 3A–C, where the fragmentation behavior of the different tilings is characterized by assessing the expected fragment size distributions following random removal of a given fraction of either passivated subunits or individually broken bonds.

The probability density function (PDF) histograms and overlaid cumulative distribution functions (CDFs) are based on the binned results of typically 10 million partially dissociated capsids per assessed distribution. They illustrate the empirical fragment size distributions *P*(*N*) at different degrees of fractional subunit and bond removal *f*_*v*_^*d*^ (and *f*_*e*_^*d*^) in the vicinity of the respective fragmentation thresholds. Note that in the case of subunit passivation and removal (*f*_*v*_^*d*^; cf. Figure 3 (middle row)) passivated subunits are excluded from the distribution, whereas for bond breakage (*f*_*e*_^*d*^; cf. Figure 3 (bottom row)) all subunits are accounted for. The graphs reveal the expected relative concentrations of fragments of various sizes upon random removal of subunits from the three distinct types of *T* = 3 tilings in Figure 1. These four distributions are computed for successively higher subunit removal fractions, *f*_*v*_^*d*^ = 10% (blue), 20% (purple), 30% (gold), and 40% (green), and are shown roughly centered about *f*_*T*_. Analogous results for random bond breakage are shown in Figure 3 (bottom row) based on the same removal values for *f*_*e*_^*d*^. Note that the only virus architecture with appreciable numbers of large clusters beyond 40% subunit removal is the kite tiling. This implies that this capsid design is much more stable than those conforming to other tiling types. This may account for the relatively rare occurrence of this tiling type in nature, and could perhaps be an indicator that this high degree of stability is not conductive to genome release.

Our predicted fragmentation threshold for HBV can be directly compared to experimental outcomes, as we have done previously in our study of the HBV capsid.^7,19^ In these experiments, the disassembly of HBV capsids was studied in which 240 C-terminal truncated HBV monomers, with a tunable fraction passivated to disable covalent bond formation, were organized in dimeric (groups of two) subunits according to a predominantly *T* = 4 surface rhombic tiling. Disassembly was triggered by addition of a mild denaturant, interrupting the remaining comparably fragile hydrophobic contacts. The maximal fraction of subunits that could be removed before capsid fragmentation occurred was identified, and agreed well with the predicted value of the fragmentation threshold (*f*_*T*_) of approximately 26%.^19^ This threshold was also observed for capsids assembled from passivated subunits only *via* the titration of mild amounts of denaturant. This strongly suggests that the theoretical predictions made here are robust against the specifics of the experimental setup.^7^ They are thus of generic interest in applications to molecular breadboard and porosity-tuned nanotechnology using other viral capsids with different lattice blueprints.

## Conclusion

A virus’ propensity for fragmentation is important for cargo release. The interplay of mutation and selection in viral evolution has resulted in capsids that balance stability, in order to provide sufficient protection for their genomic cargoes, and instability, in order to enable their timely release into the host cell environment. As we have shown here, this delicate balance hinges on capsid architecture and depends crucially on tiling type and the topology of the associated interaction network.

We demonstrate this explicitly here by computing subunit and bond fragmentation thresholds for the three *T* = 3 capsid types that correspond to the smallest nontrivial Caspar and Klug capsid layouts in virology and include architectures that are currently exploited in virus nanotechnology. One of the unexpected conclusions from our work is that virus capsids organized according to a kite tiling are a standard deviation more stable, and triangular tilings a standard deviation less stable, than rhombic tilings. This may account for the shift from triangular to predominantly rhombic tiling architectures in larger viruses in nature, suggesting that capsid stability could be a driver for the evolution of specific protein subunit architectures.

Our model is based on a number of simplifying assumptions, in particular neglect of the thermodynamic and kinetic complexities of other models. Frameworks based on reaction kinetics and thermodynamics often use (large systems of) differential equations or employ more complex molecular dynamics simulations, respectively.^25−27^ Differential equation models include subunit valency and combinatorics capturing one or a few static bond energy value(s), as well as reaction rate parameters, in order to model the kinetic approach to thermodynamic equilibrium. Molecular dynamics and Monte Carlo simulations, on the other hand, replace the static bond energy, as well as the implicit representation of subunit geometry, with explicit geometric subunit models and thermodynamic interaction potentials, as well as diffusive dynamics, in order to analyze the assembly kinetics and its long-term behavior. In contrast to these comparatively complex models, the percolation model presented here retains only the topological contributions from the subunit and bond network derived from geometric principles alone.^16,18^ The simplicity of these comparably limited, strictly topological assumptions is reflected in the ease of the percolation model’s implementation, which is much simpler than large-scale reaction kinetic models and molecular dynamics simulations. Nevertheless, this strictly topological percolation model and its prediction of the fragmentation threshold of HBV is in excellent agreement with experiment as outlined above,^7,19^ demonstrating that our approach captures essential features of capsid fragmentation. Indeed, our approach makes predictions that are testable experimentally and will enable experimentalists to detect the equivalent thresholds for any virus of interest.

We also note that the percolation threshold *p*_*c*_ studied in the broader percolation theory literature corresponds to the point of disruption of any long-range connectivity, that is, disappearance of any cluster of the order of system size, while only allowing the remaining smaller part of the system to be discontiguous. The more lenient percolation threshold *f*_*T*_ < *p*_*C*_ corresponds to long-range spanning percolation. This is comparable to the onset of fluid flow through a large-scale cluster spanning from one side of a 2D lattice medium to another, without assuming global connectivity of the lattice (here, of the remaining capsid). We argue that *f*_*T*_ is indeed the biologically and experimentally relevant quantity, as thermodynamic breakup is likely to occur much beyond the initial fragmentation threshold. This is consistent with our previous work on the fragmentation threshold of Hepatitis B virus, validated by single-particle detection methods with near single-subunit resolution, such as Charge Detection Mass Spectroscopy (CDMS) and nanofluidics.^7,19^ Such methods detect any dissociating singlets, much like the *f*_*T*_ metric itself. The observed sensitivity to the possibility of fragmented singlets or small clusters is also consistent with the size dependence of the fragmentation threshold *f*_*T*_ established here, which is not the case for the percolation threshold *p*_*c*_ in general.

The theory presented here provides a guide for applications in nanotechnology. We recently predicted and demonstrated that individual P22 VLP nanoreactors may be hierarchically assembled into ordered arrays *via* the use of small, oppositely charged linkers.^8^ Such close-packed superlattices have also been generated for cowpea clorotic mottle virus (CCMV),^28−31^ which corresponds to one of the *T* = 3 structures analyzed here. Close-packing is capable of increasing the local VLP concentration by several hundred fold, thus enhancing the catalytic activity of the cargo.^2^ Enhanced catalysis is, in part, due to the porosity of the VLPs to diffusive small molecules. The tunable presence of holes in a VLP surface is likely to increase porosity, enabling the diffusion of small molecules, and thus allowing tuning of the catalytic activity of hierarchical VLP assemblies.^2,8,32^ Such porosity tuning is already employed in the generation of porous coordination polymers for purposes of small molecule storage, separation, and catalysis.^33^ Our results enable a better understanding of how (and if) the desired porosity may be achieved in biomimetic systems.

The resistance to fragmentation and disassembly of viruses also confers many other technological advantages to VLPs. The maximal number of subunits that may be removed, *i.e.*, the number of “holes” that can be “punched” into its surfaces before it fragments, informs the use of subunit removal and replacement strategies to tune a VLP’s properties. For example, the ability to regulate the number of perforations (or modified subunits) constituting the VLPs will likely enable tuning of their elastic properties, which are commonly investigated using atomic force microscopy (AFM),^34−38^ and play a vital role in virus assembly.^11,12,39^ This type of subunit removal potentially enables particle shape to be controlled more readily by reducing the elastic moduli of the VLPs closer to regimes demonstrated to be susceptible to deformation.^34,35,40,41^ Functionalization may be possible, as the reversibility of assembly and disassembly has enabled the refilling of up to this maximum number of missing subunits with different, potentially functionalizable subunits in so-called chimeric molecular breadboards, such as those based on the HBV nucleocapsid^7^ or the P22 bacteriophage capsid.^13^ Indeed, other forms of surface functionalization of fully assembled capsids, and even simple solution additives, have already been shown to modulate the VLP elastic response.^37,38^ Array formation has also been shown to be sensitive to the net charge of the virus, which could change if mutated subunits with fewer charge moieties were substituted into the chimera.^8,32^

The extent to which missing subunits and VLP breadboard (subunit replacement) methods will be successful in tuning porosity, elasticity, charge, and other surface properties is determined, in part, by the maximal number of subunits that may be removed without inducing collapse of the entire VLP. Our results therefore not only identify drivers of viral evolution, favoring certain capsid architectures in nature, but also provide a guide for the exploitation of VLPs in virus nanotechnology, enabling better control of the biological properties of VLP-based biomimetic materials.

## Methods

### Mathematical Representation of Viral Capsid Architecture as Tilings

Blueprints of icosahedral viral capsids abide to an overarching design principle based on Archimedean lattices.^18^ We focus here on those capsid geometries in this classification that can be constructed from a single subunit type, which are the (6, 6, 6), (3, 6, 3, 6), and (3, 4, 6, 4) lattices. These geometric models refine the Caspar and Klug classification of virus architecture, in which capsids are described in terms of the triangulation number *T*, where *T* = *h*^2^ + *hk* + *k*^2^ for *h* and *k* non-negative integers. In their theory, capsids are formed from 60*T* proteins that are organized into 12 clusters of five and 10(*T* – 1) clusters of six proteins. There is only one geometric blueprint for a *T* = 3 capsid formed from 180 proteins. However, Archimedean lattice theory identifies the three distinct types of models in Figure 1 with different properties in terms of tile numbers and interaction networks.

### The Fragmentation Threshold

Subunit and bond fragmentation thresholds were computed numerically for each tiling type. After sufficient (typically 100 thousand ) replicates we observed a smooth, sigmoidal decrease in the probability that all remaining subunits are connected, *P*_*con*_, indicative of a phase transition from a connected “holey” capsid to individual fragments. We define the *f*_*T*_ as the critical percentage of subunits (or bonds) deleted at which the system is, on average, disconnected (*P*_*con*_ = 0.5). The code is available from GitHub: https://github.com/MathematicalComputationalVirology/VirusPercolationTheory.git

### Overview of Model Parameters

The parameters in Table 2 are directly relevant to our percolation theory model. The model has not otherwise been parametrized prior to numerical estimates of the final quantities of interest upon random vertex/edge deletion.
